# Addition of terlipressin to initial volume resuscitation in a pediatric model of hemorrhagic shock improves hemodynamics and cerebral perfusion

**DOI:** 10.1371/journal.pone.0235084

**Published:** 2020-07-02

**Authors:** Javier Gil-Anton, Victoria E. Mielgo, Carmen Rey-Santano, Lara Galbarriatu, Carlos Santos, Maria Unceta, Yolanda López-Fernández, Silvia Redondo, Elvira Morteruel

**Affiliations:** 1 Department of Pediatric, Pediatric Intensive Care Unit. Biocruces Bizkaia Health Research Institute, Cruces University Hospital, University of Basque Country, UPV/EHU, Barakaldo, Bizkaia, Spain; 2 Animal Research Unit, Biocruces Bizkaia Health Research Institute, Barakaldo, Bizkaia, Spain; 3 Department of Neurosurgery, Cruces University Hospital, Barakaldo, Bizkaia, Spain; 4 Department of Neurophysiology, Cruces University Hospital, Barakaldo, Bizkaia, Spain; 5 Biochemistry Laboratory, Cruces University Hospital, Barakaldo, Bizkaia, Spain; 6 Pediatric Intensive Care Unit. Biocruces Bizkaia Health Research Institute, Cruces University Hospital, Barakaldo, Bizkaia, Spain; Lundquist Institute at Harbor-UCLA Medical Center, UNITED STATES

## Abstract

Hemorrhagic shock is one of the leading causes of mortality and morbidity in pediatric trauma. Current treatment based on volume resuscitation is associated to adverse effects, and it has been proposed that vasopressors may be used in the pharmacological management of trauma. Terlipressin has demonstrated its usefulness in other pediatric critical care scenarios and its long half-life allows its use as a bolus in an outpatient critical settings. The aim of this study was to analyze whether the addition of a dose of terlipressin to the initial volume expansion produces an improvement in hemodynamic and cerebral perfusion at early stages of hemorrhagic shock in an infant animal model. We conducted an experimental randomized animal study with 1-month old pigs. After 30 minutes of hypotension (mean arterial blood pressure [MAP]<45 mmHg) induced by the withdrawal of blood over 30 min, animals were randomized to receive either normal saline (NS) 30 mL/kg (n = 8) or a bolus of 20 mcg/kg of terlipressin plus 30 mL/kg of normal saline (TP) (n = 8). Global hemodynamic and cerebral monitoring parameters, brain damage markers and histology samples were compared. After controlled bleeding, significant decreases were observed in MAP, cardiac index (CI), central venous pressure, global end-diastolic volume index (GEDI), left cardiac output index, SvO_2_, intracranial pressure, carotid blood flow, bispectral index (BIS), cerebral perfusion pressure (CPP) and increases in systemic vascular resistance index, heart rate and lactate. After treatment, MAP, GEDI, CI, CPP and BIS remained significantly higher in the TP group. The addition of a dose of terlipressin to initial fluid resuscitation was associated with hemodynamic improvement, intracranial pressure maintenance and better cerebral perfusion, which would mean protection from ischemic injury. Brain monitoring through BIS was able to detect changes caused by hemorrhagic shock and treatment.

## Introduction

Hemorrhage is the most prevalent cause of shock in injured children, being trauma one of the most common causes. Typically, hemorrhage occurs soon after injury and can produce immediate consequences, making it imperative to begin adequate resuscitative measures in initial trauma care, above all in a prehospital setting. Hemodynamic effects, which include decreases in cardiac output (CO) and blood pressure, result in a reduction in the supply of oxygen to tissues triggering an inflammatory cascade and the production of free radicals, leading to systemic capillary leak syndrome, vasoplegia and myocardial depression [1]. Pending a definitive etiology-driven approach, usually surgery, resuscitation is based on volume replacement using multiple intravenous solutions (e.g., crystalloids/colloids and hypertonic saline) [2]. Nonetheless, numerous clinical studies in adult humans and in animal models have shown that an approach involving early aggressive fluid resuscitation can have negative effects (such as dilution of coagulation factors, clot destabilization, increased bleeding, worsening of acidosis and severe hemodilution), that will reduce the oxygen supply to tissues and increase mortality. For this reason, we must develop new strategies that, avoiding the negative effects of aggressive fluid therapy, allow us to restore adequate tissue perfusion even in the initial phases of prehospital care, giving patients time to be transferred to a trauma center to receive definitive hemostatic and surgical treatment.

In relation to this issue, the use of vasopressors, in particular, noradrenaline, is starting to be assessed for the management of hemorrhagic shock, seeking to achieve the level of blood pressure needed to maintain oxygen supply [3]. The European guideline on management of major bleeding and coagulopathy following trauma recommends a restricted volume replacement strategy to achieve target blood pressure until bleeding can be controlled (Grade 1B) and administration of vasopressors in addition to fluids to maintain arterial pressure target in the presence of life-threatening hypotension (Grade 1C) [4].

Arginine vasopressin (AVP) is a stress hormone, produced in the posterior pituitary as a response to low blood pressure. Via stimulation of V1 receptors, it causes an increase in vascular resistance with a redistribution of the blood flow to the heart, brain and kidneys [5]. Its action is more intense than that of angiotensin II or noradrenalin. Given the short half-life of AVP (10–35 minutes), it needs continuous infusion and this may limit its prehospital use in cases of traumatic hemorrhagic shock. Experimental use of AVP in animal models of hemorrhagic shock is well documented [6], and it notably contributed to maintaining cerebral perfusion pressure in a porcine hemorrhage model [7]. It is not, however, free of potential adverse effects [8]. Regarding clinical practice, the results of the most promising international multicenter study on vasopressin for trauma-related shock, the Vasopressin in Refractory Traumatic Hemorrhagic Shock (VITRIS) study, are yet to be published and should help to shed light on the value of using vasopressors in resuscitation in trauma [9].

If a vasopressor drug could be used as a single dose bolus, it would reduce the hassle of transporting large volumes of fluids and need to use continuous infusion pumps, which would be of special interest for multi-casualty incidents. Terlipressin (tri-glycyl-lysine-vasopressin) (TP) is a long-acting synthetic analogue that is a more specific agonist for the V1 receptor, and can be given as a single dose and even intraosseously, which is of great interest in children, due to the difficult venous access in children. Its characteristic long half-life makes it especially interesting in situations where the patient has to wait before they are transferred to a trauma center. In previous studies, we have assessed the role of TP in other critical clinical situations, such as cardiac arrest [10] and septic shock [11] obtaining favorable hemodynamic outcomes.

The use of TP in animal models of uncontrolled hemorrhage has been associated with a tendency towards mortality reduction without increased bleeding [12]. In addition to its hemodynamic effect, associated with a better blood pressure profile, it has a notable immunomodulatory effect, reducing blood levels of proinflammatory cytokines (IL6) and increasing those of anti-inflammatory (IL10) cytokines, in turn lessening the extent of histological damage [13].

The administration of TP in combination with volume expanders may be a safe and effective therapy in the treatment of hemorrhagic shock [14], but its clinical application in this type of shock has yet to be established in children. In 2017, a meta-analysis was published [15] based on three randomized clinical trials including 224 children with shock of various etiologies who received vasopressin or TP. The analysis confirmed their hemodynamic effect (increase in blood pressure and reduction in heart rate [HR], but failed to demonstrate any benefits in terms of mortality. On the other hand, the heterogeneity of the studies included suggests the need for further research to assess their potential benefits. Indeed, a beneficial effect on mortality has been demonstrated in adult patients [16].

There is also a need for research studying potential adverse effects, such as ischemic complications involving the spleen and skin, as well as negative cardiac inotropism. For this reason, it is critically important to conduct preclinical evaluation of drugs in animal models with an age similar to that of patients to whom they may be given later. Before the use of any therapy, it is essential to estimate its repercussions at systemic, pulmonary and cerebral levels, to attempt to reduce potential any potential adverse effects during their clinical use.

Based on current data, further clinical studies designed to compare early vasopressor support plus fluid resuscitation versus fluid resuscitation alone are warranted [17]. The objective of this experiment with an infant animal model was to investigate the effect at hemodynamic and cerebral levels of a single dose of TP on initial resuscitation in a model of hemorrhagic shock without brain injury.

## Methods

This study was carried out in 17 young male pigs (10.1 ± 0.6 kg) in strict accordance with the recommendations in the Guide for the Care and Use of Laboratory Animals. The experimental protocol met European and Spanish regulations for the protection of experimental animals (2010/63/UE and RD53/2013) and was approved by the Ethics Committee for Animal Welfare of Biocruces Bizkaia Health Research Institute. Animals were obtained from a local farm authorized by the Basque Country Regulatory Agency to supply animal for research (ARRI-TURRI S.L., Galarreta, Basque Country, Spain).

### Surgical preparation

The animals were sedated with intramuscular ketamine (15 mg/kg), diazepam (2 mg/kg) and atropine (0.05 mg/kg). A face mask was placed and animals were anesthetized with 3–5% of sevoflurane and oxygen. A tracheal tube (5.0 mm ID) was inserted, through a tracheostomy, and connected to an inhalation anesthesia system with 2–3% of sevoflurane to maintain the anesthesia and analgesia during the experimental procedure. Animals were then positive pressure ventilated (VIP Bird, Bird Products Corp., Palm Springs, CA) with the following initial settings: fraction of inspired oxygen = 0.21–0.3, respiratory frequency = 20–30 breaths/minute, positive end-expiratory pressure = 4 cmH_2_O, I:E ratio = 1:2 and positive inspiratory pressure = 15–18 cmH_2_O. Deviations from acceptable blood gas values (PaO_2_ 90–110 mmHg, PaCO_2_ 35–45 mmHg and pH 7.35–7.45) were corrected by adjusting the ventilator settings. At the same time, the animals were paralyzed using a continuous IV infusion of atracurium besylate (3 mg/kg/h). Continuous three-lead ECG monitoring was established in all animals.

A thermodilution arterial catheter (5 Fr, PiCCO Plus, Pulsion, München, Germany) was inserted into the femoral artery to monitor MAP and HR, to measure CI and to obtain arterial blood samples for blood gas analysis (PaO_2_, PaCO_2_, pH, base excess, SaO_2_), lactate, hemoglobin (Hb) and hematocrit (GEM Premier 4000, Instrumentation Laboratory Company, Lexington, MA). In addition, a 7 Fr three-lumen catheter was inserted into the internal jugular vein to inject cold saline, monitor central venous pressure (CVP), obtain blood samples for blood gas analysis and biochemical analysis and perform blood withdrawal and volume expansion. Blood temperature, measured with a thermistor at the thermodilution arterial catheter tip, was maintained at 38–39°C using an overhead warmer throughout the experiment.

### Experimental design

After surgery, animals were allowed to stabilize for 20 min. Once a steady state was achieved and basal data had been gathered, hypovolemic shock was induced by the withdrawal of blood over 30 min until MAP was less than 45 mmHg for 30 min (Hemo). At that point, animals were randomly assigned to:

- Normal saline (NS) Group (n = 8): animals received an intravenous bolus of 30 mL/kg of normal saline over 30 min.
- Terlipressin (TP) Group (n = 8): animals received a single dose of 20 mcg/kg of TP (Glypressin, Ferring Pharmaceuticals, Saint-Prex, Switzerland) and, immediately afterwards, an intravenous bolus of 30 mL/kg of normal saline over 30 min.

**Fig 1:**
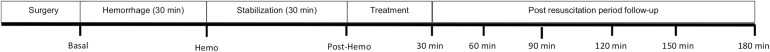
Timeline for the experiment.

The experimental design is outlined in Fig 1.

### Measurements

At several time points (Fig 1), blood samples were drawn from the femoral artery and jugular vein to measure blood gases, base excess and lactate, including electrolytes. MAP, HR, CVP and temperature were monitored (IntelliVue MP50, Phillips, The Netherlands) (Fig 1).

A thermodilution method was used to assess CO at five points during the experimental period (basal, hemo, 30 min, 90 min and 180 min). Briefly, 5 ml of cold saline (< 8ºC) was injected into the central venous catheter to be dispersed volumetrically and thermally within the pulmonary and cardiac volumes. This volume of distribution is named the intrathoracic volume. When the thermal signal reaches the thermistor at the tip of the femoral catheter, a temperature difference is detected and a dissipation curve is generated. Then, the monitor uses specific algorithms to determine the cardiac output, by integrating the area under the curve of the arterial pressure vs. time trace. The global end-diastolic volume index (GEDI) was obtained by advanced analysis of the thermodilution curve.

Subsequently, the following variables were calculated:

Cardiac index (CI) (L/min/m^2^) = CO/body surface
Systemic vascular resistance index (SVRI) (mmHg/ml.kg^-1^.min^-1^) = (MAP-CVP)/CI
Left cardiac output index (LCOI) (Kg.m/m^2^) = CI x MAP x 0.0136
Arterial (venous) oxygen content (Ca(v)O_2_) (O_2_ ml/dl) = (Sa(v)O_2_ x Hb x 1.39/100) + (Pa(v)O_2_ x 0.003)
Oxygen delivery (OD) (O_2_ ml/kg/min) = CaO_2_ x CI
Oxygen consumption (VO_2_) (O_2_ ml/kg/min) = (CaO_2_ –CvO_2_) x CI
Fractional tissue oxygen extraction (FTOE) = (SaO_2_ –SvO_2_)/SaO_2,_ where SaO_2_ is arterial oxygen saturation and SvO_2_ is venous oxygen saturation.

The right common carotid blood flow was measured, with an ultrasonic flow probe (Transonic Systems Inc., NY), as a proxy for cerebral blood flow. For continuous monitoring of intracranial pressure (ICP) during the experiment, a cranial window was opened using a mini-drill, to place an intraparenchymal fiber optic sensor (Codman ICP EXPRESS Monitoring System; Codman Neuro, Raynham, MA, USA), over the left coronal suture. It was fixed to the skull using a screw designed for this purpose. ICP values were recorded continuously throughout the experimental period. The CPP was calculated using a standard formula (CPP = MAP − ICP).

The level of electrical activity in the brain was assessed using a bispectral index (BIS). This was measured by placing a bilateral sensor on the frontal-temporal region coupled to the BIS Vista™ monitor (Aspect Medical Systems, Newton, Massachusetts), which recorded data until the end of the experiment.

### Sample collection and histological analysis

Standard complete blood counts and coagulation studies were performed at basal point, after inducing hypovolemia and at 90 and 180 min during the follow-up period. The concentrations of neuronal-specific enolase (NSE) and S100β protein in cerebrospinal samples taken at the end of the experiment were measured using specific ELISA kits (DRG International, Inc., Springfield, NJ).

At the end of the experiment, animals were sacrificed with an overdose of anesthetic (6% of sevoflurane, 1.5 mg/kg of besilate of atracurium and 300 mg/kg of potassium chloride). Postmortem, the brain was removed, fixed (4% formalin) and divided into different regions: cortex, thalamus and hippocampus. Samples of the aforementioned regions were embedded in paraffin wax and sectioned at 5 μm. Slides from each section were stained with hematoxylin-eosin and analyzed by light microscopy. The histological examinations were carried out by a pathologist who was blinded to group allocation. Brain injury was scored using a semi-quantitative scoring system.

Pathological features of brain injury (inflammation, hemorrhage, edema and infarction) were each scored on a 0- to 3-point scale: 0 corresponding to no injury; and 1, 2, and 3 to injury to mild, moderate, and severe injury across the field. A total of 20 fields were analyzed. The presence of more than five necrotic cells per field was considered to indicate neuronal necrosis (summing a score of 1 for each field with ≥ 5 necrotic cells, yielded a necrosis score, range: 0–20).

### Statistical analysis

Data were analyzed using JMP statistical discovery software (version 8, SAS Institute Inc., North Carolina). One-way analysis of variance (ANOVA) was performed to assess time point differences in gas exchange, cardiovascular parameters and systemic oxygenation and perfusion as a function of group. Comparisons of results at all time points were performed by two-way repeated-measures ANOVA as a function of group and time. Comparison of measured values with basal and hemo time points were assessed with Student’s paired t test. Simple linear correlation analysis was used to assess the relationship between various cardiovascular and cerebral parameters. A p < 0.05 was considered statistically significant. Values are expressed as mean ± SD.

## Results

The volume of blood extracted in each group was similar, 30±2 ml/kg, considered to be equivalent to 45–50% of the blood volume. One animal died during blood extraction and 16 were randomized.

### Hemodynamics

Controlled hemorrhage resulted in a significant decrease in MAP, CI, CVP, GEDI and LCOI with a significant increase in SVRI and HR in both groups (Table 1).

**Table 1:** Hemodynamic parameters.

		Basal	Hemo	30 min	60 min	90 min	120 min	150 min	180 min
**HR** bpm	NS	171±29	229±24^&^	200±20^#^	197±30^#^	200±32^#^	199±30^#^	195±31^#^	189±31^#^
	TP	166±18	228±29^&^	204±27^#^	202±25^#^	201±23^#^	196±19^#^	195±25^#^	189±22^#^
**MAP**^$^ mmHg	NS	72±11	38±5^&^	53±5^#^	43±4^#^	39±4	37±3	35±5^#^	31±5^#^
	TP	67±6	37±2^&^	65±8*^#^	54±6*^#^	51±6*^#^	45±5*^#^	43±5*^#^	38±4*
**CI**^$^ L/min/m^2^	NS	4.6±0.8	2.1±0.1^&^	3.2±0.4^#^	2.7±0.4^#^	2.6±0.3^#^	2.3±0.3	2.3±0.3	2.1±0.3
	TP	4.5±0.6	2.2±0.3^&^	3.4±0.5^#^	3.1±0.5^#^	2.9±0.4 ^#^	2.8±0.5*^#^	2.7±0.5*^#^	2.6±0.4*^#^
**LCOI**^$^ Kg.m/m^2^	NS	4.6±1.1	1.1±0.2^&^	2.2±0.3^#^	1.6±0.4^#^	1.4±0.2^#^	1.2±0.2	1.1±0.2	0.9±0.2
	TP	4.2±0.7	1.1±0.2^&^	3.0±0.6*^#^	2.3±0.5*^#^	2.0±0.4*^#^	1.7±0.4*^#^	1.6±0.4*^#^	1.4±0.3*^#^
**SVRI**^$^ mmHg/ml.kg^-1^.min^-1^	NS	1168±263	1333±15^&^	1157±217	1177±230	1091±180^#^	1099±204^#^	1058±213^#^	1017±222^#^
	TP	1085±159	1219±15^&^	1361±227^#^	1324±274	1280±229	1160±215	1109±182	1019±157^#^
**CVP** mmHg	NS	7±1	5±1^&^	6±1	5±1	5±1	5±1	5±1	5±1
	TP	7±1	5±2^&^	6±1	6±2	6±1	6±1	6±1	6±1
**GEDI**^$^ ml/m^2^	NS	369±29	199±28^&^	242±31^#^	229±37^#^	226±35^#^	210±41	211±38	210±42
	TP	364±39	213±37^&^	283±50^#^	273±57^#^	244±29^#^	248±47^#^	241±44^#^	240±32^#^

(^$^) p<0.05 two-factor ANOVA

(^&^) p<0.05 Hemo vs. Basal time point in the same group

(^#^) p<0.05 treatment time points vs. Hemo in the same group

(*) p<0.05 One-Factor ANOVA. NS (normal saline); TP (terlipressin); HR (heart rate); MAP (mean arterial blood pressure); CI (cardiac index); LCOI (Left cardiac output index); SVRI (systemic vascular resistance index); CVP (central venous pressure; GEDI (global end-diastolic volume index).

**Fig 2:**
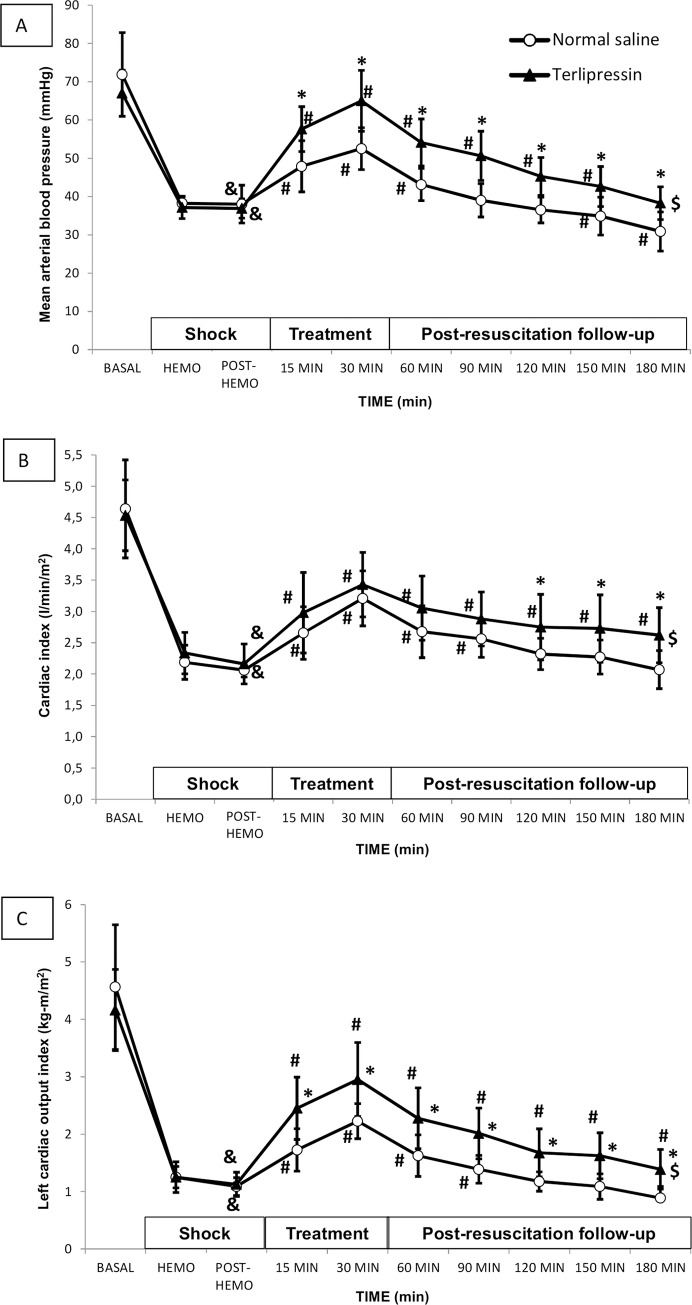
(A) Mean arterial blood pressure (MAP), (B) cardiac index (CI) and (C) left cardiac output index (LCOI) in normal saline group (white circles) and terlipressin group (black triangles). Data are expressed as mean and SD. ($) p<0.05 two-factor ANOVA; (^&^) p<0.05 Hemo vs. basal time point in the same group; (^#^)p<0.05 treatment time points vs. Hemo in the same group; (*) p<0.05 One Factor ANOVA.

Regarding treatment, tachycardia induced by hemorrhage slightly decreased in both groups, without significant differences between both groups. Animals receiving TP maintained significantly higher mean MAP than animals receiving NS alone during treatment and follow-up time points until the end of the experiment. Post-hemorrhage, MAP in the TP group, was significantly higher than in controls up to 150 minutes and remained above 40 mmHg. In the NS group, MAP fell to below 40 mmHg after 60 minutes, becoming significantly lower than post-hemorrhage levels by the final minutes (Fig 2A).

In the TP group, CI remained significantly higher than immediately post-hemorrhage throughout the observation period and higher than in the NS group, overall and especially in the last hour (Fig 2B). Logically, given the results observed for MAP and CI, LCOI in animals receiving TP was significantly higher than post-hemorrhage at all subsequent time points and also higher than values in those receiving NS comparing point-by-point (Fig 2C).

**Fig 3:**
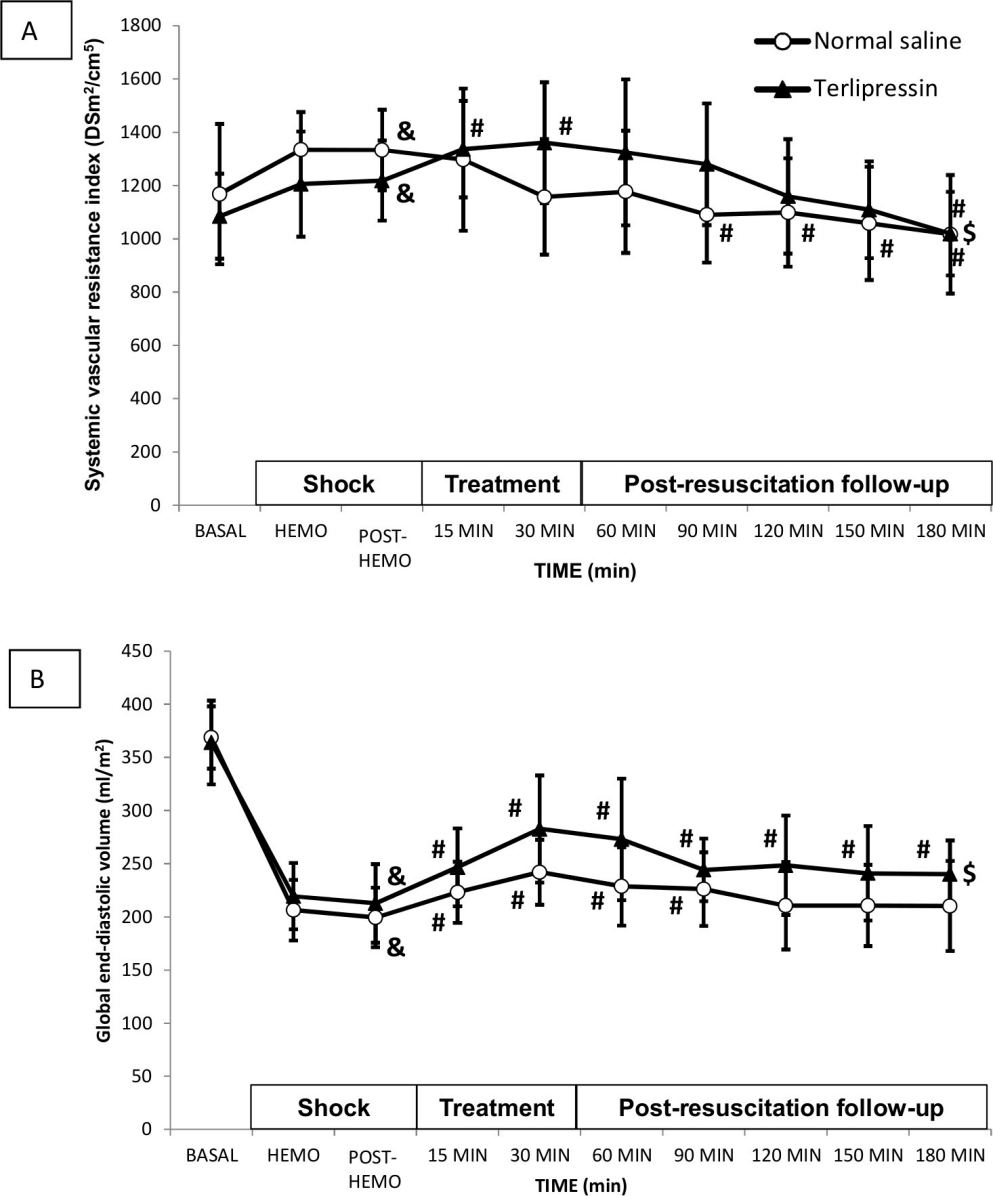
(A) Systemic vascular resistance index (SVRI) and (B) global end-diastolic volume index (GEDI) in normal saline group (white circles) and terlipressin group (black triangles). Data are expressed as mean and SD. ($) p<0.05 two-factor ANOVA; (^&^) p<0.05 Hemo vs. basal time point in the same group; (^#^)p<0.05 treatment time points vs. Hemo in the same group.

In the TP group, there was a short-term increase (30 min) in SVRI after the hemorrhage, with more normal values than in the NS group. In the NS group, SVRI significantly decreased over the 90 minute time point. That pronounced decrease was not observed until the last follow-up time point (180 minutes) in the TP group (Fig 3A).

Regarding the assessment of blood volume, differences in CVP between treatment groups were not significant. Furthermore GEDI in the TP group was significantly higher than after hemorrhage at all subsequent time points and also higher than values in the NS group (Fig 3B).

### Blood gases, electrolytes and hematology

Exsanguination led to lactic acidosis that improved with treatment, though pH had not recovered to basal values by the end of follow-up, with no significant differences between groups. SvO_2_ fell after the hemorrhage in both groups, with a tendency to recover more initially in the NS group and at the end of the experiment in the TP group, but differences did not reach significance. Arterial oxygen content decreased slightly in relation to the anemia developed after treatment, while venous oxygen content fell earlier, at the time of hemorrhagic shock, largely related to the increase in oxygen extraction. Although differences between groups were not large, we note that oxygen delivery was better in the TP group in the last 90 minutes of follow-up, in line with the improved CI.

Hemorrhage led to hyperkalemia, which improved over the last hour of follow-up, and hyponatremia and hypochloremia, without significant differences between groups. Regarding hematological parameters, hemoglobin and hematocrit decreased after volume replacement, again without significant differences between groups. Further, no differences were found between treatments administered in white blood cell count or clotting parameters. These results are shown in S1 Table.

Table 2 lists the results of the blood gas, oxygen transport and hematological analyses.

**Table 2:** Blood tests results.

		Basal	Hemo	30 min	60 min	90 min	120 min	150 min	180 min
**pH**	NS	7.46±0.05	7.34±0.09^&^	7.35±0.07	7.38±0.07	7.39±0.05	7.39±0.03	7.39±0,04	7.38±0.04
	TP	7.43±0.05	7.34±0.07^&^	7.35±0.06	7.36±0.03	7.37±0.03	7.38±0.02	7.39±0.04	7.39±0.02
**Lactate** mmol/l	NS	1.0±0.4	2.8±0.8^&^	1.9±0.5^#^	1.7±0.5^#^	1.6±0.5^#^	1.7±0.7^#^	1.7±0.8^#^	2.0±1.0
	TP	0.9±0.4	2.6±0.7^&^	1.8±0.7^#^	1.6±0.6^#^	1.5±0.6^#^	1.5±0.5^#^	1.6±0.6^#^	1.8±0.7^#^
**SvO**_**2**_^$^ %	NS	81±9	35±12^&^	62±7^#^	51±10^#^	43±10^#^	45±9^#^	46±8^#^	43±12
	TP	83±6	41±16^&^	52±13	44±16	47±23	50±13	57±17	52±17
**CaO**_**2**_ O_2_ ml/dl	NS	11.0±1.1	10.7±1.0	8.4±1.1	8.9±0.7	8.8±1.2	9.0±1.0	8.8±0.7	8.7±1.1
	TP	11.4±1.7	10.7±1.0	8.6±1.0	9.1±1.4	9.7±1.4	9.2±1.2	9.3±1.1	9.2±1.3
**CvO**_**2**_ O_2_ ml/dl	NS	8.7±1.2	3.7±1.2	5.2±1.2	4.4±0.7	3.7±0.9	4.0±0.8	4.0±0.7	3.6±0.8
	TP	9.0±1.3	4.3±2.0	4.4±1.0	3.8±1.7	4.3±2.2	4.6±1.5	5.3±1.8	4.8±1.9
**VO**_**2**_ O_2_ ml/kg/min	NS	41±14	61±16	43±9	48±8	53±12	48±8	45±6	43±12
	TP	38±14	60±21	60±19*	67±21*	66±34	53±14	46±17	47±14
**OD** O_2_ ml/kg/min	NS	212±52	92±13	111±14	97±11	92±9	87±8	82±7	75±10
	TP	224±70	98±14	123±16	117±24	120±25*	107±23*	107±25*	101±21*
**FTOE**^$^	NS	0.20±0.08	0.65±0.12^&^	0.39±0.07	0.50±0.09	0.57±0.10	0.56±0.08	0.54±0.07	0.58±0.11
	TP	0.18±0.06	0.60±0.18^&^	0.49±0.13	0.59±0.17	0.55±0.24	0.50±0.13	0.44±0.17	0.48±0.17
**K**^**+**^ mmol/l	NS	3.5±0.5	4.2±0.8^&^	3.3±1.0^#^	3.9±0.8	4.3±0.9	4.6±1.0	4.7±0.9	5.3±1.0^#^
	TP	3.5±0.5	4.3±0.7^&^	3.7±0.9^#^	4.1±0.9	4.6±0.9	4.6±1.1	4.9±0.8^#^	5.4±0.9^#^
**Na**^**+**^ mmol/l	NS	142±2	137±4^&^	141±2^#^	140±2^#^	137±7	139±1	139±3	138±2
	TP	143±3	140±2^&^	142±2^#^	141±3	139±3	141±3	140±4	138±3^#^
**Cl**^**-**^ mmol/l	NS	114±3	109±5^&^	115±3^#^	114±3^#^	111±7	112±3^#^	113±4^#^	112±3^#^
	TP	114±4	112±3^&^	116±2^#^	115±3^#^	112±4	114±3^#^	114±4	112±3
**Ca**^**++**^ mmol/l	NS	1.08±0.15	1.16±0.12	1.06±0.13^#^	1.14±0.12	1.13±0.15	1.12±0.12	1.03±0.10^#^	1.04±0.10^#^
	TP	1.12±0.15	1.17±0.11	1.04±0.10^#^	1.10±0.16	1.20±0.23	1.05±0.12^#^	1.05±0.15^#^	1.02±0.16^#^
**Hb** (g/dl)	NS	9.8±1.7	9.4±2.7			7.3±0.9^#^			7.1±0.8^#^
	TP	9.6±1.2	8.9±0.6			7.9±0.7^#^			7.5±0.7^#^
**Hto** (%)	NS	30±6	30±9			23±2^#^			23±2^#^
	TP	30±4	29±1			25±3^#^			24±2^#^

($) p<0.05 two-factor ANOVA

(^&^) p<0.05 Hemo vs. basal time point in the same group

(^#^)p<0.05 treatment points vs. Hemo in the same group

(*) p<0.05 One Factor ANOVA. NS (normal saline); TP (terlipressin); CaO_2_ (arterial oxygen content); CvO_2_ (venous oxygen content); OD (oxygen delivery); VO_2_ (oxygen consumption); FTOE (fractional oxygen extraction); Hto (hematocrit); Hb (hemoglobin).

### Neurological status

The experimental data from neurological monitoring are reported in Table 3.

**Table 3:** Results of the neurological monitoring.

		Basal	Hemo	30 min	60 min	90 min	120 min	150 min	180 min
**ICP** mmHg	NS	8±2	6±1^&^	10±2^#^	10±2^#^	10±2^#^	9±2^#^	9±2^#^	8±2^#^
	TP	7±3	5±1^&^	8±2^#^	8±2^#^	8±2^#^	8±1^#^	8±2^#^	8±1^#^
**CPP**^$^ mmHg	NS	62±12	30±7^&^	42±6^#^	33±4	29±5	26±4^#^	25±7^#^	22±7^#^
	TP	62±3	32±4^&^	59±9*^#^	47±7*^#^	43±7*^#^	38±5*^#^	35±5*	31±4*
**Qcar**^$^ ml/min	NS	82±28	32±18^&^	50±21^#^	39±19^#^	34±14^#^	32±13	31±13	27±11
	TP	74±28	34±11^&^	55±14^#^	44±13^#^	46±14^#^	42±15^#^	43±12^#^	37±9
**BIS**^$^	NS	35±7	27±8^&^	28±6	27±12	23±11	25±9	28±8	25±8
	TP	36±6	22±10^&^	42±10*^#^	37±9*	35±6*	42±13*	43±5*^#^	41±10*^#^

(^$^) p<0.05 two-factor ANOVA

(^&^) p<0.05 Hemo vs. basal timepoint in the same group

(^#^) p<0.05 treatment points vs. Hemo in the same group

(*) p<0.05 One Factor ANOVA. NS (normal saline); TP (terlipressin); ICP (intracranial pressure); CPP (cerebral perfusion pressure); Qcar (carotid blood flow); BIS (Bispectral index).

**Fig 4:**
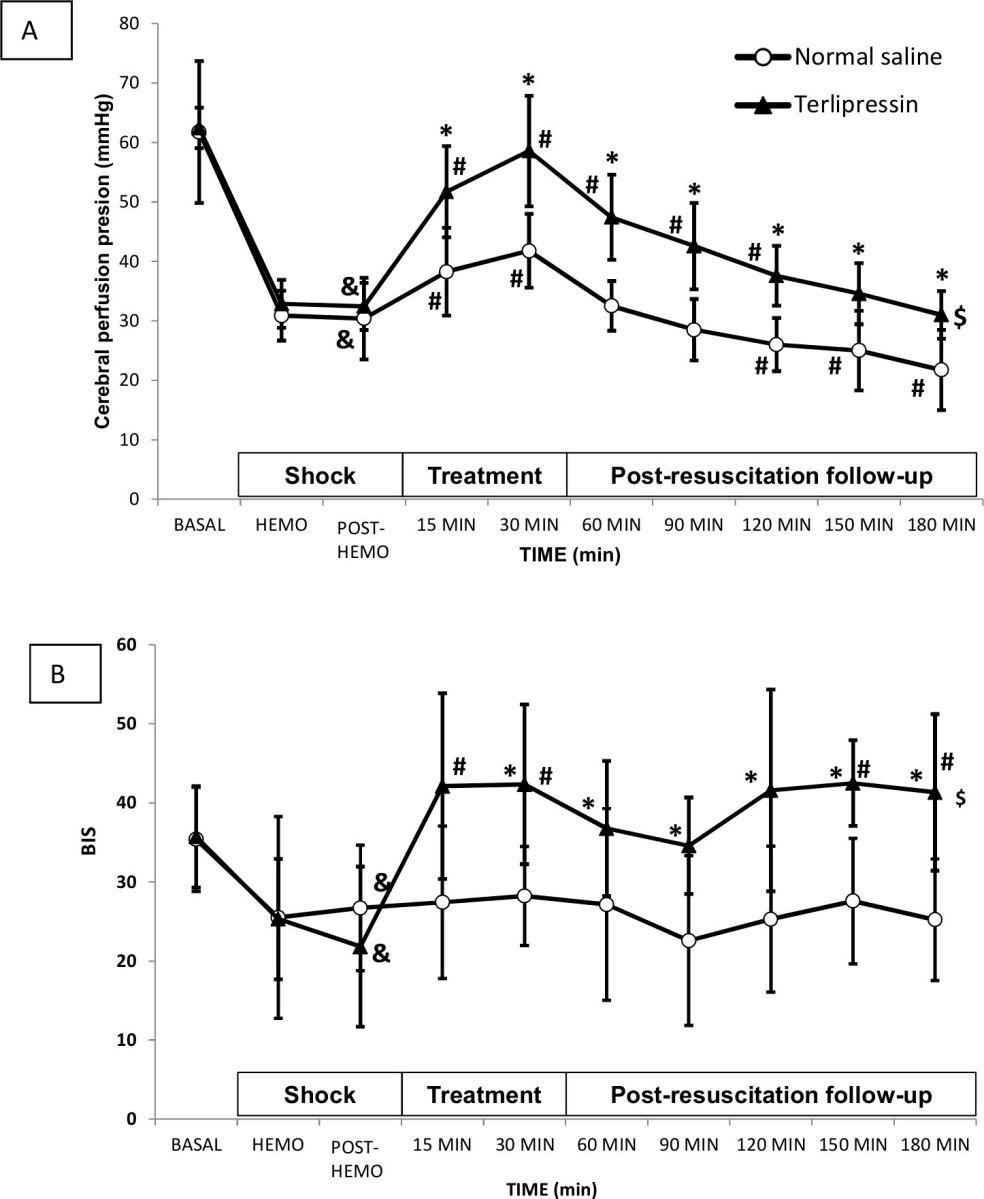
(A) Cerebral perfusion pressure (CPP) and (B) bispectral index (BIS) in the normal saline group (white circles) and terlipressin group (black triangles). Data are expressed as mean and SD. ($) p<0.05 two-factor ANOVA; (^&^) p<0.05 Hemo vs. basal time point in the same group; (^#^) p<0.05 treatment time points vs. Hemo in the same group; (*) p<0.05 One Factor ANOVA.

Hemorrhage led to significant decreases from basal point in ICP, carotid blood flow, BIS and CPP in both groups. ICP recovered with treatment in both groups, with no significant differences between them at any point during follow-up. The most relevant differences were found in the analysis of CPP. Namely, in the TP group, pressure was significantly higher than in the NS group at all time points and from 30 to 120 minutes remained significantly higher than the immediately after hemorrhage. Furthermore, it remained above 40 mmHg up to 90 minutes. In contrast, in the NS group, CPP was significantly lower than immediately post-hemorrhage from 90 minutes onwards (Fig 4A).

Carotid blood flow initially improved with treatment in both groups, but this improvement was sustained after 90 minutes with TP, resulting in an overall significant difference. The correlation between carotid flow and CI (0.71, p<0.0001) was good. Regarding BIS, significant recovery after hemorrhage was observed in the TP group at 30, 150 and 180 minutes, and values were higher than in the NS group at each time point (Fig 4B).

About markers of brain damage in CSF, neither of the parameters measured differed significantly in the TP group at the end of the experiment: concentrations of NSE of 1.7±0.8 mcg/l vs 2.7±3.7 mcg/l in controls and S100β of 2.7±0.9 vs 2.7±1.9 mcg/l in controls. Similarly, histological analysis based on data on necrosis, edema, inflammation, hemorrhage and infarction in the thalamic regions, occipital cortex and hippocampus did not reveal any significant differences, with mild edema, inflammation and hemorrhage in both groups (mean score less than 1 in all parameters) without infarction. The mean necrosis score was 8±4 in the TP group and 6±3 in the NS group, without significant differences between them (necrosis score range: 0–20).

## Discussion

### Hemodynamics

Exsanguination led to the expected hemodynamic changes: decreases in MAP, CVP and CI and increases SVRI and HR. Our analysis focused on assessing and comparing the changes observed within 3 hours after exsanguination with the two resuscitation regimens. The group treated with TP had a better blood pressure profile, and importantly, pressure remained above the threshold of 40 mmHg for longer, which is key to being able to maintain CPP during patient transfer while awaiting definitive care. This effect on blood pressure maintenance over time has previously been described in rats, lasting for 75 minutes [13]. In our series, we cannot rule out an additional effect of TP counteracting vasodilation of inhaled anesthesia, but in a clinical scenario, sedation/anesthesia would be necessary, and hence, this pharmacologic vasodilatation would be frequently present.

The effect on arterial pressure was accompanied by a better pattern of cardiac output. To explain this positive result for both MAP and output, we analyzed the behavior of the two hemodynamic determinants that could be affected by TP. Regarding systemic resistances, it has been described that the endogenous release of catecholamines is able to maintain them high with respect to baseline for some time [5]. In our study, the addition of the vasoconstrictor was associated with longer maintenance of the values obtained in response to the aggression. In another experimental porcine model, no variations were observed in SRVI attributable to the addition of TP, although it was given at a dose of 15 mcg/kg and compared to hypertonic saline [18].

Concerning preload, CVP did not differ between groups, although the low reliability of this measure for assessing blood volume has been widely described [19]. On the other hand, a more specific parameter for measuring blood volume, namely, GEDI, does reflect a better outcome for animals treated with TP; specifically, they had significantly higher GEDI and this elevation was maintained throughout the experiment compared to the fall after exsanguination. As a consequence, we believe that improvement in the preload through the effect on venous capacitance would have a leading role in the effect of the resistances on hemodynamics, since it is the only factor that is able to increase MAP and CI at the same time. The good correlation between these two parameters in our study supports this hypothesis. This role of vasopressors in the mobilization of fluid from venous capacitance vessels to the central circulation has been previously described in animal trials [20] and septic patients [21].

This overall beneficial hemodynamic effect is demonstrated in LCOI, which was significantly higher in the TP group, as has previously been described in an adult experimental model [22], both with Ringer lactate and with TP. In our model, the combination of TP and volume expansion was associated with a greater increase than that observed using volume loading alone.

This hemodynamic profile obtained with a single dose of TP is particularly interesting given that its use would be a rapid and easily implementable action, which would be a great advantage in the context of sudden mass casualties due to bombings or other types of attack which have been increasing in recent years [23].

### Oxygen, electrolyte and blood gas levels

Firstly, we have not found a correlation between treatment with TP and lactic acidosis. It is known that oxygen extraction increases due to hemorrhage and remains high after volume resuscitation [24]. Our study shows a more favorable profile for the TP group in terms of better venous saturation and CI, which would be more relevant than the actual maintenance of cardiac output in terms of any tissue-protecting effect.

Our strategy was not associated with changes in coagulation function, or even in platelet count, which would support this strategy of restricted volume expansion to avoid coagulopathy related to hemodilution [25].

### Neurological status

In animals treated with TP, there was better maintenance of CPP, based on greater MAP values, as there was no significant change in ICP. This effect was maintained over time and has clinical relevance, given that the pressure stayed >40 mmHg for 90 minutes and significantly higher than the post-hemorrhage value for 2 hours. This contribution of TP to the maintenance of CPP has been previously described in an adult animal model without trauma brain injury and no basal ICP increase [22] while in porcine polytrauma models, the maintenance of CPP has been correlated with a reduction in brain mitochondrial dysfunction [26], which would provide protection against secondary brain injury.

The pathophysiological effect of TP on venous compliance might optimize initial hemodynamics and consequently prevent neurological damage associated with hemorrhagic shock, helping to maintain CPP [27] and consequently brain oxygen supply.

Basal BIS was low, as would be expected, due to sedation and significantly decreased with the hemorrhage. Treatment with TP achieved significant improvements in BIS compared to values in the NS group. TP was associated with a statistically significant recovery in BIS following the blood loss at 30, 150 and 180 minutes and, at all times, BIS was significantly better than in the NS group, which hardly changed with treatment. This positive effect of the use of vasoconstrictors on cerebral perfusion, reflected in BIS, has previously been reported with vasopressin in hemorrhagic resuscitation in an adult porcine model [28] and opens up the possibility of cerebral monitoring with BIS during trauma care provision.

We did not observe differences in either histological analysis or neuronal markers, which may be explained by the short observation period prior to sample collection. A more specific analysis of the water balance, oxidative damage and apoptosis in an adult porcine model demonstrated a protective effect of TP [22] in a similar observation period.

With respect to cerebrospinal fluid markers of brain injury, neuron-specific enolase is an isoenzyme present in the neural and neuroendocrine cells and it is considered to be the most specific enzymatic marker of brain damage. S100β protein is an acidic, dimeric, calcium-binding protein, mostly found in the cytoplasm of astrocytes and Schwann cells, used as a marker of central nervous system damage. Elevated levels NSE (2.1 mcg/l) and S100β (1.1 mcg/l) have been described in a sham group in neonatal models 6 hours after hypoxic insult [29]. In our study, we did not have such a long observation period or a sham group, and hence, we are only able to draw conclusions concerning the lack of differences between treatment groups.

### Limitations

The experimental model itself has limitations compared to real trauma, given that it involves controlled hemorrhage without lesions associated with brain injury. The logical requirements of animal welfare make it necessary to use anesthetics, which have effects at hemodynamic and neurological levels. In that sense, we lacked a sham group for comparison. About tissue damage, the study was restricted to the first 3 hours of care, and we did not assess cell ischemia or microcirculation. Furthermore, the results in these animals cannot be directly extrapolated to humans. The inflammatory response, mechanisms of coagulation, and vasopressin receptors are different in swine [30]. In terms of the monitoring, we underline that BIS has been developed for use in humans and it may not be possible to extrapolate values obtained in swine to humans, given the differences in head shape. Lastly, in this study, only male animals were used.

## Conclusions

The addition of one dose of TP to initial resuscitation with fluids resulted in a hemodynamic improvement (preload, cardiac output and blood pressure). In cases of hemorrhagic shock, the use of a vasopressor with a long half-life would help maintain the hemodynamic improvement for the time needed for a patient to reach hospital where they could receive definite hemostatic and surgical treatment. The addition of TP to initial resuscitation was significantly associated with an improvement in CPP and higher carotid blood flow, without significant changes in intracranial pressure in a model without brain injury. All these factors are associated with the preservation of cerebral blood flow, which implies protection against ischemic injury; however, the implications of these results should be tested in a brain injury/polytrauma model. Early biochemical and histopathological analyses did not reveal any significant differences between the treatments, and therefore, we are not able to establish whether there was any benefit or harm in terms of additional tissue damage associated with TP over a short follow-up. Our findings need to be confirmed in longer-term studies. Brain monitoring using BIS is able to detect changes caused by induced hemorrhagic shock and treatment response. All these findings are experimental and should be confirmed in pediatric patients.

## Supporting information

S1 Table Hematological results. (^#^) p<0.05 treatment points vs. Hemo in the same group. NS (normal saline); TP (terlipressin); PT (prothrombin time); INR (international normalized ratio).
(DOCX)

S1 Raw data (PDF)
