# Supplementary material for: Addition of terlipressin to initial volume resuscitation in a pediatric model of hemorrhagic shock improves hemodynamics and cerebral perfusion
Source: PLoS One. 2020 Jul 2;15(7):e0235084. doi: 10.1371/journal.pone.0235084 (PMC7332053; doi:10.1371/journal.pone.0235084)
Supplement: S1 Table — (#) p<0.05 treatment points vs. Hemo in the same group. NS (normal saline); TP (terlipressin); PT (prothrombin time); INR (international normalized ratio). (DOCX) [file pone.0235084.s001.docx]

**Table S1. Hematological results**

|  |  | **Basal** | **Hemo** | **90 min** | **180 min** |
| --- | --- | --- | --- | --- | --- |
| **Fibrinogen**  (mg/dl) | NS | 462±68 | 428±50 | 379±59 ^#^ | 391±71 |
|  | TP | 526±134 | 490±119 | 426±78^#^ | 433±75^,#^ |
| **INR** | NS | 0.93±0.04 | 0.92±0.04 | 0.96±0.05 | 0.98±0.04^#^ |
|  | TP | 0.95±0.04 | 0.92±0.04 | 0.97±0.06^#^ | 1.00±0.08^#^ |
| **PT** (s) | NS | 10.9±0.5 | 10.7±0.3 | 11.2±0.7 | 11.5±0.4^#^ |
|  | TP | 11.1±0.5 | 10.7±0.5 | 11.3±0.7 | 11.6±1.0^#^ |
| **PT** (%) | NS | 116±9 | 118±7 | 110±12 | 105±8^#^ |
|  | TP | 111±8 | 117±8 | 106±12 | 102±16^#^ |
| **Cell Red**  (x10^6^/ul) | NS | 5.5±1.1 | 5.4±1.6 | 4.2±0.5^#^ | 4.1±0.4^#^ |
|  | TP | 5.3±0.6 | 5.2±0.4 | 4.6±0.6^#^ | 4.4±0.5^#^ |
| **Leukocyte** (x10^6^/ul) | NS | 8.2±2.8 | 13.1±6.6 | 10.4±4.6 | 7.5±3.9^#^ |
|  | TP | 7.0±3.4 | 9.2±7.3 | 8.0±7.6 | 7.8±8.0 |
| **Platelet** (x10^3^/ul) | NS | 405±140 | 378±158 | 404±87 | 412±76 |
|  | TP | 362±77 | 353±71 | 325±69^#^ | 326±62^#^ |

(^#^) p<0.05 treatment points vs. Hemo same group. NS (normal saline); TP (terlipressin); PT (prothrombin time); INR (international normalized ratio).
