# Supplementary material for: Addition of terlipressin to initial volume resuscitation in a pediatric model of hemorrhagic shock improves hemodynamics and cerebral perfusion
Source: PLoS One. 2020 Jul 2;15(7):e0235084. doi: 10.1371/journal.pone.0235084 (PMC7332053; doi:10.1371/journal.pone.0235084)
Supplement: S1 Raw data — (PDF) [file pone.0235084.s002.pdf]

S2. DATA SET

HEMODYNAMICS

	MAP (mmHg)							
	BASAL	HEMO	30 MIN	60 MIN	90 MIN	120 MIN	150 MIN	180 MIN
NS	53	32	50	38	32	33	33	29
	83	38	56	42	39	36	33	30
	64	35	55	45	37	34	30	28
	75	39	53	46	41	40	37	36
	64	37	43	40	36	34	32	29
	74	37	48	38	38	34	33	25
	76	37	54	47	43	39	35	29
	86	49	61	49	46	42	46	41
TP	67	32	65	56	53	46	46	46
	71	38	53	44	39	38	34	34
	74	36	70	61	56	52	46	40
	65	38	70	53	49	46	40	38
	55	39	57	47	51	45	45	36
	70	36	61	53	44	38	36	33
	63	40	78	60	58	48	47	37
	71	36	66	59	55	49	47	42

	HR (bpm)							
	BASAL	HEMO	30 MIN	60 MIN	90 MIN	120 MIN	150 MIN	180 MIN
NS	141	173	176	142	133	142	149	140
	176	241	231	239	227	230	223	223
	214	224	198	189	174	165	148	142
	193	230	188	188	199	199	191	195
	182	235	213	219	214	217	203	194
	147	246	212	201	219	207	208	192
	127	247	172	181	208	212	222	208
	185	235	209	220	224	217	219	216
TP	143	160	148	149	155	155	158	160
	170	243	229	208	189	178	168	166
	171	230	192	202	195	200	191	185
	142	235	198	195	201	199	193	193
	161	225	201	191	201	205	238	232
	175	232	217	223	220	205	197	183
	163	254	233	223	230	210	210	195
	199	248	215	223	220	212	203	198

	CI (l/min/m2)							
	BASAL	HEMO	30 MIN	60 MIN	90 MIN	120 MIN	150 MIN	180 MIN
NS	3,95	1,90	2,38	2,43	2,43	2,48	2,48	2,02
	4,24	2,24	2,88	2,07	2,07	1,93	1,93	1,69
	4,31	2,17	3,40	3,40	2,48	2,38	2,00	2,07
	5,48	2,02	3,36	2,74	2,74	2,50	2,50	2,40
	5,95	2,02	3,71	3,07	3,07	2,50	2,50	2,57
	3,86	1,98	3,28	2,40	2,40	2,10	2,10	1,70
	4,15	2,05	3,65	2,73	2,73	2,60	2,60	2,10
	5,16	2,11	3,00	2,57	2,57	2,07	2,07	2,00
TP	4,55	2,07	3,00	3,10	2,76	2,76	2,86	3,02
	5,64	2,14	3,17	2,88	2,88	2,57	2,57	2,69
	4,10	1,74	3,76	3,76	2,71	2,71	2,43	2,38
	3,88	2,05	2,95	2,51	2,51	2,54	2,54	2,27
	4,19	2,86	4,26	3,77	3,77	3,86	3,86	3,35
	4,26	2,02	3,10	2,40	2,40	2,00	2,00	2,00
	4,70	2,21	4,05	3,19	3,19	2,93	2,93	2,42
	4,95	2,21	3,14	2,81	2,81	2,63	2,63	2,84

	SVRI (mmHg/ml.kg^{-1}.min^{-1})							
	BASAL	HEMO	30 MIN	60 MIN	90 MIN	120 MIN	150 MIN	180 MIN
NS	931	1175	1444	922	856	872	936	830
	1453	1250	1304	1505	1312	1285	1119	1230
	1076	1181	1080	963	1066	907	960	849
	1022	1462	1119	1227	1110	1087	1023	1031
	766	1304	797	937	833	928	864	777
	1480	1457	1050	1133	1066	1104	1066	988
	1407	1248	1052	1262	1174	1138	953	990
	1209	1589	1413	1463	1308	1469	1546	1439
TP	1090	1119	1653	1292	1505	1071	1119	1058
	907	1269	1162	1082	916	964	964	832
	1347	1380	1424	1169	1502	1296	1350	1142
	1258	1327	1517	1528	1400	1324	1072	1128
	898	922	939	849	955	808	787	740
	1144	1343	1447	1629	1264	1319	1239	1079
	1004	1231	1265	1481	1305	1064	1064	992
	1033	1158	1477	1563	1392	1430	1278	1184

	CVP (mmHg)							
	BASAL	HEMO	30 MIN	60 MIN	90 MIN	120 MIN	150 MIN	180 MIN
NS	7	5	7	6	6	7	6	7
	7	4	5	5	6	5	5	5
	7	6	6	6	6	6	7	7

	6	4	5	4	4	5	5	5
	7	5	5	5	5	6	5	5
	7	3	6	5	5	5	5	5
	6	4	4	4	4	4	4	4
	9	5	6	6	5	5	5	5
TP	7	5	4	6	6	6	6	6
	7	5	5	6	6	7	6	7
	7	6	5	6	6	6	6	6
	6	3	5	6	5	5	5	5
	9	11	8	10	6	6	6	5
	8	4	5	4	4	5	5	6
	8	5	7	7	6	5	8	7
	6	4	5	5	5	5	5	5

	**LCOI (kg.m/m^2)**							
	BASAL	HEMO	30 MIN	60 MIN	90 MIN	120 MIN	150 MIN	180 MIN
NS	2,8	0,9	1,6	1,2	1,1	1,1	1,2	0,8
	4,8	1,2	2,1	1,2	1,1	1,0	0,8	0,7
	3,8	1,1	2,4	2,2	1,3	1,1	0,8	0,8
	5,7	1,1	2,4	1,8	1,6	1,3	1,3	1,2
	5,2	1,0	2,1	1,7	1,5	1,2	1,1	1,0
	3,7	1,0	2,2	1,3	1,2	1,0	0,9	0,6
	4,5	1,0	2,6	1,7	1,7	1,5	1,3	0,9
	6,0	1,4	2,4	1,9	1,6	1,2	1,3	1,1
TP	4,3	1,0	2,7	2,3	2,2	1,6	1,8	1,9
	5,4	1,1	2,2	1,7	1,5	1,3	1,3	1,2
	3,8	0,9	3,7	3,1	2,1	1,8	1,6	1,3
	3,5	1,1	2,4	1,8	1,7	1,6	1,4	1,2
	3,2	1,6	3,3	2,4	2,6	2,4	2,3	1,6
	4,0	1,0	2,6	1,7	1,4	1,0	1,0	0,9
	4,3	1,2	4,0	2,9	2,5	1,8	1,9	1,2
	4,8	1,1	2,7	2,3	2,1	1,9	1,7	1,8

	**GEDI (ml/m^2)**							
	BASAL	HEMO	30 MIN	60 MIN	90 MIN	120 MIN	150 MIN	180 MIN
NS	348	257		288	288	290	290	281
	345	202	210	179	179	186	186	169
	336	200	264	264	243		212	226
	400	202	262	240	240	231	231	231
	381	186	240	224	224	202	202	233
	355	163	233	213	213	193	193	193
	365	210	285	235	235	210	210	203
	420	175	200	186	186	161	161	145

TP	431	257	321	321	283	283		286
	345	181	224	229	229	236	236	236
	340	183	343	343	252		231	231
	340	212	254	241	241	234	234	217
	379	276	333	345		329	329	279
	326	181	221	195	195	190	190	188
	414	223	314	274	274	258	258	249
	337	188	251	235	235	209	209	235

BLOOD GASES, ELECTROLYTES AND HEMATOLOGY

	CaO_2 (O_2 ml/dl)							
	BASAL	HEMO	30 MIN	60 MIN	90 MIN	120 MIN	150 MIN	180 MIN
NS	9,8	9,8	8,9	9	7,7	9,2	9,1	8,8
	12,9	12,5	10,7	10,6	11,4	11,1	10,2	11,3
	10,6	11,1	8,4	8,8	9,2	9,6	9,2	8,7
	9,8	9,2	7,2	8,1	7,9	7,7	7,9	7,8
	12,1	10,8	7,9	8,6	8,4	8,7	8,5	8,6
	10,4	11,2	7,8	8,4	8,1	8,8	8,4	8,5
	10,7	10,4	8	8,7	8,8	8,6	8,6	7,8
	11,4	10,9	8,1	8,8	8,6	8,5	8,4	8,1
TP	11,8	11,5	9,7	9,5	10,5	9,5	10,2	10,7
	11,0	9,2	7,6	7,2	7,3	6,9	6,8	6,4
	10,0	11,0	7,9	9,4	9,0	10,6	9,9	9,5
	11,7	10,9	8,4	8,7	9,5	9,3	9,8	10,0
	9,0	9,3	7,2	7,6	8,3	8,2	8,6	8,2
	11,1	10,4	9,7	9,4	10,6	9,1	9,5	9,7
	11,6	11,2	8,7	9,2	10,6	10,0	9,4	9,2
	15,0	12,1	9,5	11,7	11,7	9,9	9,9	9,7

	CvO_2 (O_2 ml/dl)							
	BASAL	HEMO	30 MIN	60 MIN	90 MIN	120 MIN	150 MIN	180 MIN
NS	9,2	3,7	6,1	5,1	3,3	4,4	4,8	3,3
	10,3	3,5	7,7	3,5	3,8	3,4	3,9	3,6
	7,9	6,1	5,5	5,7	5,6	5,7	4,7	4,5
	7,7	4,1	4,5	4,2	3,7	3,4	3,7	3,5
	10,6	3,4	4,6	4,7	3,3	3,8	3,6	2,8
	8,3	4,3	4,5	3,7	3,8	4,1	4,0	3,0
	7,3	2,7	4,8	4,2	3,8	3,8	4,6	5,0
	8,0	1,9	4,0	4,0	2,5	3,2	2,8	3,1
TP	8,9	4,2	5,6	6,4	7,5	5,4	7,8	7,7
	9,3	3,7	5,4	2,4	3,3	4,0	4,0	3,4
	9,9	8,7	5,4	6,2	6,7	7,0	6,4	6,3
	8,0	4,2	3,8	3,1	2,8	3,1	6,8	6,0

	6,8	3,8	3,5	3,2	5,8	3,8	4,6	4,0
	8,7	2,6	3,1	2,3	1,7	2,8	2,3	2,0
	9,0	4,5	4,3	4,0	4,2	4,7	4,0	3,2
	11,4	2,4	3,9	2,5	2,1	6,0	6,1	5,8

	**VO_2 (O_2 ml/kg/min)**							
	BASAL	HEMO	30 MIN	60 MIN	90 MIN	120 MIN	150 MIN	180 MIN
NS	58	48	28	40	44	50	43	46
	27	84	37	62	66	63	51	54
	40	46	41	40	37	39	38	36
	30	44	38	45	48	45	44	44
	43	62	52	51	61	51	51	63
	25	54	43	45	43	39	37	37
	41	63	47	48	54	50	41	23
	64	83	55	55	69	48	51	44
TP	33	66	51	41	35	50	31	39
	48	49	30	58	45	31	27	33
	11	17	40	51	27	41	36	32
	50	56	55	58	74	64	32	37
	29	68	67	71	42	73	67	61
	47	66	85	72	89	53	61	64
	37	63	77	71	88	66	68	63
	49	92	75	112	124	44	42	48

	**OD (O2 ml/kg/min)**							
	BASAL	HEMO	30 MIN	60 MIN	90 MIN	120 MIN	150 MIN	180 MIN
NS	157	78	89	92	77	96	91	75
	230	117	130	92	99	90	82	80
	192	101	120	114	95	96	77	75
	226	78	102	93	91	81	82	79
	303	91	123	112	100	91	89	93
	153	88	102	80	81	74	70	58
	177	85	117	94	96	90	89	65
	259	101	107	99	98	77	76	71
TP	212	104	122	124	121	115	132	136
	260	83	101	87	81	74	68	72
	162	81	124	151	103	121	101	95
	186	91	101	89	105	97	102	93
	162	114	131	123	142	136	143	118
	199	88	126	95	107	77	80	81
	233	106	151	126	145	126	119	96
	375	115	128	142	152	112	111	119

	FTOE							
	BASAL	HEMO	30 MIN	60 MIN	90 MIN	120 MIN	150 MIN	180 MIN
NS	0,37	0,62	0,32	0,44	0,57	0,52	0,47	0,62
	0,12	0,72	0,29	0,67	0,67	0,70	0,62	0,68
	0,21	0,45	0,34	0,35	0,39	0,41	0,49	0,48
	0,13	0,56	0,38	0,48	0,53	0,56	0,53	0,56
	0,14	0,68	0,42	0,46	0,60	0,56	0,57	0,68
	0,16	0,61	0,43	0,56	0,53	0,53	0,53	0,65
	0,23	0,74	0,40	0,51	0,57	0,56	0,46	0,36
	0,25	0,82	0,51	0,55	0,71	0,62	0,67	0,62
TP	0,16	0,64	0,42	0,33	0,29	0,44	0,23	0,29
	0,19	0,60	0,30	0,66	0,55	0,41	0,40	0,46
	0,07	0,21	0,32	0,34	0,26	0,34	0,36	0,34
	0,27	0,61	0,55	0,65	0,71	0,66	0,31	0,40
	0,18	0,59	0,51	0,58	0,30	0,54	0,47	0,52
	0,24	0,75	0,68	0,76	0,84	0,69	0,76	0,79
	0,16	0,59	0,51	0,57	0,60	0,53	0,58	0,65
	0,13	0,80	0,59	0,79	0,82	0,39	0,38	0,40

	**Hb (g/dl)**				**Hto (%)**			
	**BASAL**	**HEMO**	**90 MIN**	**180 MIN**	**BASAL**	**HEMO**	**90 MIN**	**180 MIN**
NS	8,8	8,2	7,0	7,0	28,5	27,0	22,9	24,1
	10,4	10,0	9,4	8,8	31,9	31,1	28,0	27,4
	9,2	8,5	7,5	7,4	25,5	27,6	24,1	23,5
	8,2	7,5	6,3	6,2	26,0	23,8	20,1	20,1
	9,7	9,2	7,1	7,0	30,9	30,4	23,7	23,0
	9,5	7,7	7,2	7,2	30,2	24,4	22,8	22,8
	8,6	8,7	6,9	6,6	26,5	26,9	21,9	20,8
	13,6	15,7	6,9	6,5	43,7	50,9	23,3	21,1
TP	9,4	8,9	8,6	8,1	29,5	28,0	27,1	25,8
	10,6	8,1	6,3	6,0	33,6	26,0	20,1	19,4
	9,5	9,3	7,9	7,9	28,8	28,8	24,6	24,4
	9,5	9,6	7,7	7,6	29,7	29,6	24,2	24,4
	7,5	8,4	7,9	7,1	25,5	29,4	27,3	24,7
	10,1	8,7	8,1	7,8	31,5	28,3	25,9	25,0
	8,8	8,7	7,7	7,6	27,5	28,2	25,2	24,7
	11,5	9,8	8,7	8,2	36,8	31,0	29,0	26,4

	**Lactate (mmol/l)**							
	BASAL	HEMO	30 MIN	60 MIN	90 MIN	120 MIN	150 MIN	180 MIN
NS	0,7	2,5	2,2	2,0	2,7	3,3	3,4	4,2
	0,9	2,7	1,9	1,4	1,2	1,0	1,0	1,2
	0,9	2,3	1,8	1,3	1,2	1,3	1,3	1,4

	0,8	2,7	1,5	1,6	1,6	1,5	1,6	1,6
	0,8	3,8	2,6	2,4	2,1	1,9	1,9	2,4
	0,7	1,7	1,3	1,2	1,2	1,2	1,2	1,4
	1,2	2,3	1,4	1,3	1,3	1,4	1,5	2,1
	2,0	4,0	2,5	2,3	1,8	1,7	1,4	1,6
TP	0,6	1,9	1,0	0,7	0,7	0,8	0,9	1,1
	0,9	3,4	2,7	2,3	2,3	2,2	2,4	2,3
	1,0	2,7	1,7	1,7	1,5	1,4	1,5	1,4
	0,7	1,6	1,2	1,1	1,2	1,1	1,3	1,3
	0,8	2,3	1,7	1,4	1,2	1,6	1,7	2,5
	0,9	3,7	3,0	2,2	2,2	2,3	2,4	2,9
	0,7	2,2	1,2	1,2	1,2	1,2	1,1	1,3
	1,8	3,1	2,2	2,2	2,0	1,4	1,3	1,5

	**Na+ (mmol/l)**							
	BASAL	HEMO	30 MIN	60 MIN	90 MIN	120 MIN	150 MIN	180 MIN
NS	143	137	138	140	137	136	137	135
	141	127	144	142	122	139	142	140
	145	140	143	143	141	139	140	140
	141	136	140	137	137	137	134	135
	141	139	142	141	140	138	138	136
	143	141	143	142	143	140	142	139
	141	140	140	139	139	139	139	139
	139	138	141	139	140	140	138	138
TP	141	139	139	142	138	137	140	136
	144	140	144	143	144	145	147	143
	149	142	142	141	140	140	139	138
	141	138	142	137	140	141	142	140
	141	138	139	138	138	137	137	138
	146	144	144	144	141	142	141	139
	140	139	141	140	132	139	134	134
	143	140	146	143	141	143	142	139

	**K+ (mmol/l)**							
	BASAL	HEMO	30 MIN	60 MIN	90 MIN	120 MIN	150 MIN	180 MIN
NS	4,4	5,7	5,5	5,7	6,4	6,8	6,8	7,4
	3,3	5,1	2,6	3,3	4,7	4,1	3,8	4,7
	3,0	3,8	3,0	3,4	3,9	4,6	4,6	4,7
	3,2	3,7	3,1	4,1	4,1	4,2	4,6	5,1
	3,7	3,9	2,9	3,6	3,9	4,5	4,9	5,8
	3,0	3,7	3,3	3,8	3,9	4,4	4,4	5,2
	3,4	3,8	3,5	4,1	4,3	4,4	4,5	5,0
	3,7	3,5	2,4	3,0	3,4	3,6	4,0	4,3

TP	4,3	5,6	5,2	5,1	5,9	6,0	5,6	6,5
	3,2	4,3	3,4	3,6	4,0	4,0	4,5	4,2
	2,7	3,6	3,1	4,0	3,8	4,0	4,7	4,8
	3,5	3,6	3,6	4,0	4,6	4,6	4,6	5,2
	3,9	4,9	5,0	5,8	5,7	6,3	6,3	6,8
	3,3	3,6	3,1	3,3	4,0	3,7	4,3	4,9
	3,6	4,2	3,3	4,0	5,2	5,0	5,3	5,7
	3,6	4,2	2,7	3,2	3,9	3,5	3,8	4,7

	**Cl- (mmol/l)**							
	BASAL	HEMO	30 MIN	60 MIN	90 MIN	120 MIN	150 MIN	180 MIN
NS	115	106	110	112	109	108	108	108
	111	99	114	113	96	111	116	112
	116	109	115	117	113	111	113	114
	113	109	115	114	111	111	116	112
	112	110	115	113	114	112	111	111
	119	116	121	119	119	117	119	117
	113	112	114	113	112	113	114	113
	109	110	114	112	113	113	111	111
TP	113	110	115	116	113	113	114	110
	115	115	117	118	118	116	117	117
	121	111	115	113	112	113	111	111
	113	115	118	115	115	116	119	117
	112	109	112	111	110	109	109	110
	117	113	117	119	114	118	115	114
	111	110	114	114	105	112	109	108
	110	109	117	113	112	116	115	110

	**Ca++ (mmol/l)**							
	BASAL	HEMO	30 MIN	60 MIN	90 MIN	120 MIN	150 MIN	180 MIN
NS	0,91	1,05	1,01	0,94	0,97	1,05	0,96	0,94
	1,22	1,23	1,20	1,26	1,38	1,25	0,99	1,10
	0,99	1,30	1,15	1,12	1,20	1,30	1,07	1,00
	0,96	1,06	0,87	1,12	0,99	0,96	1,00	1,04
	1,01	1,03	0,89	1,00	0,98	0,97	0,92	0,94
	1,02	1,10	1,07	1,12	1,08	1,11	0,99	1,05
	1,12	1,15	1,15	1,24	1,19	1,15	1,09	1,03
	1,37	1,32	1,16	1,28	1,27	1,17	1,24	1,24
TP	1,15	1,23	1,02	0,85	1,12	1,05	0,98	1,11
	1,08	1,07	1,02	0,95	0,95	0,80	0,81	0,70
	0,86	1,20	0,87	1,13	1,04	1,02	1,25	1,09
	1,08	0,98	1,03	1,05	1,11	1,07	0,91	0,94
	1,04	1,11	0,97	1,16	1,04	1,13	1,02	0,91

	1,09	1,22	1,19	1,07	1,27	1,04	1,11	1,12
	1,34	1,30	1,07	1,25	1,49	1,24	1,23	1,11
	1,29	1,28	1,13	1,33	1,58	1,06	1,07	1,21

	**SvO2 (%)**							
	BASAL	HEMO	30 MIN	60 MIN	90 MIN	120 MIN	150 MIN	180 MIN
NS	62,8	36,6	68,6	58,0	43,9	47,9	53,0	37,8
	88,5	28,4	71,1	33,2	33,1	29,7	37,5	31,6
	80,1	54,8	66,5	66,2	61,8	59,5	51,8	53,3
	88,3	45,4	63,5	53,3	47,8	45,4	47,5	45,3
	87,0	32,4	59,1	55,1	39,5	45,4	43,2	32,4
	84,6	38,8	59,4	44,8	48,4	47,9	47,8	36,4
	78,4	25,8	61,0	49,6	44,1	44,9	54,7	66,0
	75,8	16,7	49,2	45,2	28,6	38,0	32,9	38,1
TP	85,3	35,9	57,7	67,7	70,7	56,4	75,9	70,8
	82,1	39,2	68,1	32,4	43,9	59,4	61,0	54,3
	94,1	79,7	70,8	67,2	75,4	66,5	65,1	67,2
	74,2	38,7	46,3	36,3	29,2	34,0	72,1	61,8
	83,4	40,9	50,2	41,8	70,8	45,7	52,6	48,7
	76,5	23,9	31,9	23,6	15,9	30,5	23,6	21,1
	84,6	41,1	49,9	44,2	40,3	48,1	42,6	34,6
	85,9	32,2	41,6	35,4	30,4	62,0	62,8	61,0

	**pH**							
	BASAL	HEMO	30 MIN	60 MIN	90 MIN	120 MIN	150 MIN	180 MIN
NS	7,57	7,53	7,46	7,50	7,49	7,45	7,45	7,45
	7,46	7,25	7,28	7,31	7,35	7,37	7,40	7,39
	7,47	7,41	7,38	7,40	7,40	7,41	7,42	7,43
	7,40	7,34	7,34	7,37	7,39	7,36	7,37	7,33
	7,46	7,30	7,29	7,32	7,35	7,38	7,36	7,37
	7,44	7,30	7,38	7,37	7,36	7,36	7,38	7,34
	7,49	7,36	7,39	7,43	7,42	7,40	7,43	7,41
	7,40	7,26	7,27	7,31	7,33	7,35	7,34	7,35
TP	7,42	7,36	7,35	7,37	7,36	7,36	7,32	7,35
	7,40	7,23	7,26	7,31	7,34	7,41	7,45	7,40
	7,48	7,40	7,43	7,39	7,37	7,38	7,38	7,40
	7,46	7,38	7,39	7,41	7,40	7,40	7,43	7,42
	7,45	7,41	7,38	7,38	7,41	7,37	7,37	7,38
	7,38	7,26	7,28	7,35	7,36	7,36	7,36	7,37
	7,45	7,37	7,36	7,37	7,39	7,39	7,42	7,41
	7,32	7,32	7,33	7,33	7,33	7,36	7,38	7,39

NEUROLOGICAL STATUS

	ICP (mmHg)							
	BASAL	HEMO	30 MIN	60 MIN	90 MIN	120 MIN	150 MIN	180 MIN
NS	10	7	9	9	9	9	8	8
	6	6	11	10	10	10	9	8
	10	8	12	14	13	11	11	10
	8	4	8	8	7	8	7	6
	10	8	11	11	12	12	11	10
	6	6	7	7	7	7	7	6
	6	5	9	9	9	9	8	8
	7	6	9	9	9	9	8	9
TP	6	5	8	9	10	9	10	9
	6	6	9	10	8	7	6	7
	8	4	6	7	8	8	7	7
	7	4	7	6	6	7	7	7
	9	5	9	8	8	7	8	8
	2	4	5	4	5	6	6	6
	10	5	9	10	11	10	10	9
	9	6	8	9	9	8	9	8

	Qcar (ml/min)							
	BASAL	HEMO	30 MIN	60 MIN	90 MIN	120 MIN	150 MIN	180 MIN
NS	69	33	59	46	41	38	37	30
	31	1	17	9	10	8	6	6
	70	21	23	20	23	26	22	22
	91	27	56	43	35	29	27	26
	106	61	81	67	55	46	38	31
	96	45	47	31	29	28	35	27
	72	31	52	43	35	36	36	31
	123	42	66	54	49	46	50	47
TP	40	13	44	36	44	33	47	44
	113	43	54	50	46	45	42	40
	70	41	70	66	68	68	57	45
	48	25	38	26	27	27	26	26
	45	29	43	37	48	41	43	35
	81	35	51	36	28	24	24	24
	74	41	80	54	56	52	52	39
	118	43	60	50	53	51	53	49

	BIS							
	BASAL	HEMO	30 MIN	60 MIN	90 MIN	120 MIN	150 MIN	180 MIN
NS	34	24	32	39	24	32	33	25
	38	31	29	34	36	32	34	35
	49	32	25	12	10	10	17	21

	30	31	38	40	29	32	26	21
	30	14	19	13	11	20	21	14
	35	19	24	19	15	18	23	26
	32	36	32	33	34	34	39	35
TP	41	30	34	35	37	41	48	54
	30	13	50	32	30	31	33	36
	33	11	54	53	46	65	41	40
	35	15	41	38	36	40	43	42
	44	34	42	31	29	28	45	37
	30	21	27	30	31	45	46	27
	38	27	47	43	37	41	43	54

	**CPP (mmHg)**							
	BASAL	HEMO	30 MIN	60 MIN	90 MIN	120 MIN	150 MIN	180 MIN
NS	44	25	41	29	23	24	25	21
	76	32	45	32	29	26	24	22
	54	27	43	31	24	23	19	18
	67	35	45	38	34	32	30	30
	51	27	32	29	24	22	21	19
	67	31	41	31	31	27	26	19
	58	22	35	30	26	21	17	12
	77	44	52	40	37	33	38	33
TP	61	27	57	47	43	37	36	37
	64	32	44	34	31	31	28	27
	69	32	64	54	48	44	39	33
	58	34	63	47	43	39	33	31
	60	32	59	46	42	37	35	32
	62	31	52	45	36	31	28	25
	61	40	73	56	53	42	41	31
	62	31	57	49	44	39	37	33

HEMATOLOGICAL RESULTS

	**FIBRINOGEN (mg/dl)**				**INR**			
	**BASAL**	**HEMO**	**90 MIN**	**180 MIN**	**BASAL**	**HEMO**	**90 MIN**	**180 MIN**
NS	428,7	424,0	349,2	390,8	0,89	0,87	1,01	0,97
	600,0	477,0	480,0	527,0	0,91	0,97	0,94	0,93
	426,0	389,5	340,7	351,2	0,88	0,90	0,92	0,97
	392,2	379,9	323,0	324,3	0,92	0,91	0,90	0,95
	441,2	403,6	364,6	359,9	0,92	0,91	0,91	0,97
	490,4	517,1	444,6	441,0	1,01	0,94	0,99	1,02
	456,9	406	352,5	341,1	0,96	0,96	1,03	1,03
TP	751,0	713,7	536,8	537,7	0,98	0,94	1,04	1,12

	399,8	369,6	325,2	343,8	0,97	0,98	0,96	1,00
		493,1	437,2	448,6	0,98	0,90	0,96	0,95
	471,4	440,1	408,5	422,6	0,87	0,86	0,87	0,89
	427,1	365,3	329,3	329,3	0,95	0,95	1,03	1,07
	489,8	513,7	458,4	471,2	0,97	0,89	0,96	0,97
	618,3	535,7	483,5	478,1	0,96	0,94	0,99	1,00

	**PT (seg)**				**PT %**			
	**BASAL**	**HEMO**	**90 MIN**	**180 MIN**	**BASAL**	**HEMO**	**90 MIN**	**180 MIN**
NS	10,7	10,4	12,0	11,6	123,2	126,0	97,3	104,3
					120,0	108,0	114,0	116,0
	10,2	10,4	10,7	11,2	126,0	124,4	117,7	107,6
	10,7	10,5	10,4	11,0	118,7	123,5	126,0	112,1
	10,7	10,6	10,6	11,2	117,7	119,8	119,8	107,6
	11,7	10,9	11,5	11,8	98,5	114,2	102,1	96,8
	11,1	11,1	11,9	12	110	110	95,1	93,4
TP	11,7	11,2	12,4	13,3	103,1	113,2	91,1	78,5
	11,2	11,4	11,1	11,6	107,6	104,0	109,5	100,5
		10,4	11,1	11,0	115,5	124,4	109,5	111,5
	10,1	10,0	10,1	10,3	126,0	126,0	126,0	126,0
	11,0	11,0	12,0	12,4	112,1	112,1	93,4	87,2
	11,3	10,3	11,1	11,2	105,9	126,0	110,0	107,9
	11,1	10,9	11,5	11,6	110	114,2	102,1	100,3

	**Leukocytes (x 10^3/ul)**				**Cell red (x 10^6/ul)**			
	**BASAL**	**HEMO**	**90 MIN**	**180 MIN**	**BASAL**	**HEMO**	**90 MIN**	**180 MIN**
NS	6,53	17,08	14,68	5,32	5,15	4,80	4,04	4,22
	13,97	14,42	15,94	16,34	5,96	5,83	5,25	5,14
	6,05	10,33	7,99	6,49	4,48	4,75	4,16	4,08
	6,59	25,44	5,74	9,7	5,08	4,61	3,88	3,90
	10,59	13,95	10,97	6,43	5,39	5,23	4,05	3,93
	7,61	6,23	6,33	5,68	5,14	4,17	3,84	3,87
	5,6	4,23	16,13	4,21	4,90	4,96	4,00	3,79
	8,72	13,44	5,6	6,25	8,03	9,16	4,23	3,9
TP	4,28	22,06	21,62	23,53	5,50	5,12	4,98	4,73
	10,55	5,01	2,31	1,30	4,35	4,44	3,39	3,29
	6,13	19,65	18,37	16,97	5,20	5,17	4,39	4,38
	11,68	3,67	6,11	5,01	5,28	5,20	4,22	4,25
	10,41	4,17	5,54	5,28	5,12	5,86	5,41	4,89
	3,59	6,17	3,05	2,94	5,50	4,84	4,45	4,33
	3,54	6,40	4,25	3,46	5,15	5,20	4,65	4,57
	5,54	6,27	2,82	3,84	6,42	5,38	5,06	4,60

	Platelets (x 10^3/ul)			
	BASAL	HEMO	90 MIN	180 MIN
NS	628	592	526	528
	414	396	339	404
	306	302	299	318
	202	188	325	328
	569	531	449	469
	309	273	350	350
	411	531	443	427
	397	208	504	470
TP	348	311	306	319
	222	296	267	264
	299	278	249	244
	377	449	412	417
	372	276	277	309
	485	404	373	361
	393	435	424	399
	399	376	286	293
